# Supplementary material for: An allele-resolved nanopore-guided tour of the human placental methylome
Source: Nat Commun. 2025 Nov 24;16:10358. doi: 10.1038/s41467-025-65337-3 (PMC12644668; doi:10.1038/s41467-025-65337-3)
Supplement: Supplementary file 1 — Supplementary Information [file 41467_2025_65337_MOESM1_ESM.pdf]

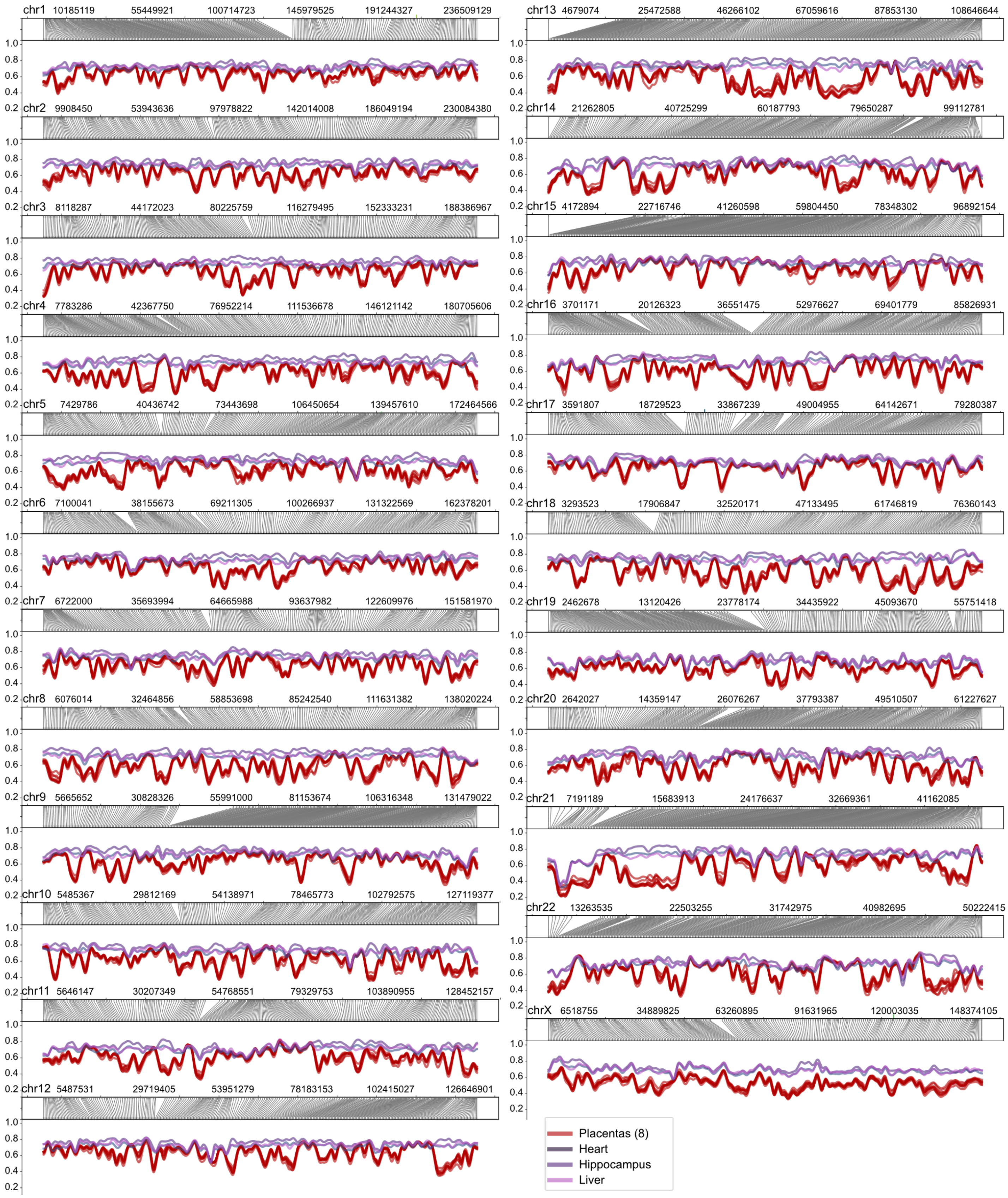

Supplemental Figure 1: Methylation profiles for placentas (red) as compared to other tissues (heart, hippocampus, liver). Each panel shows genome coordinate space translated to CpG coordinate space, chromosomes are not scaled by length.

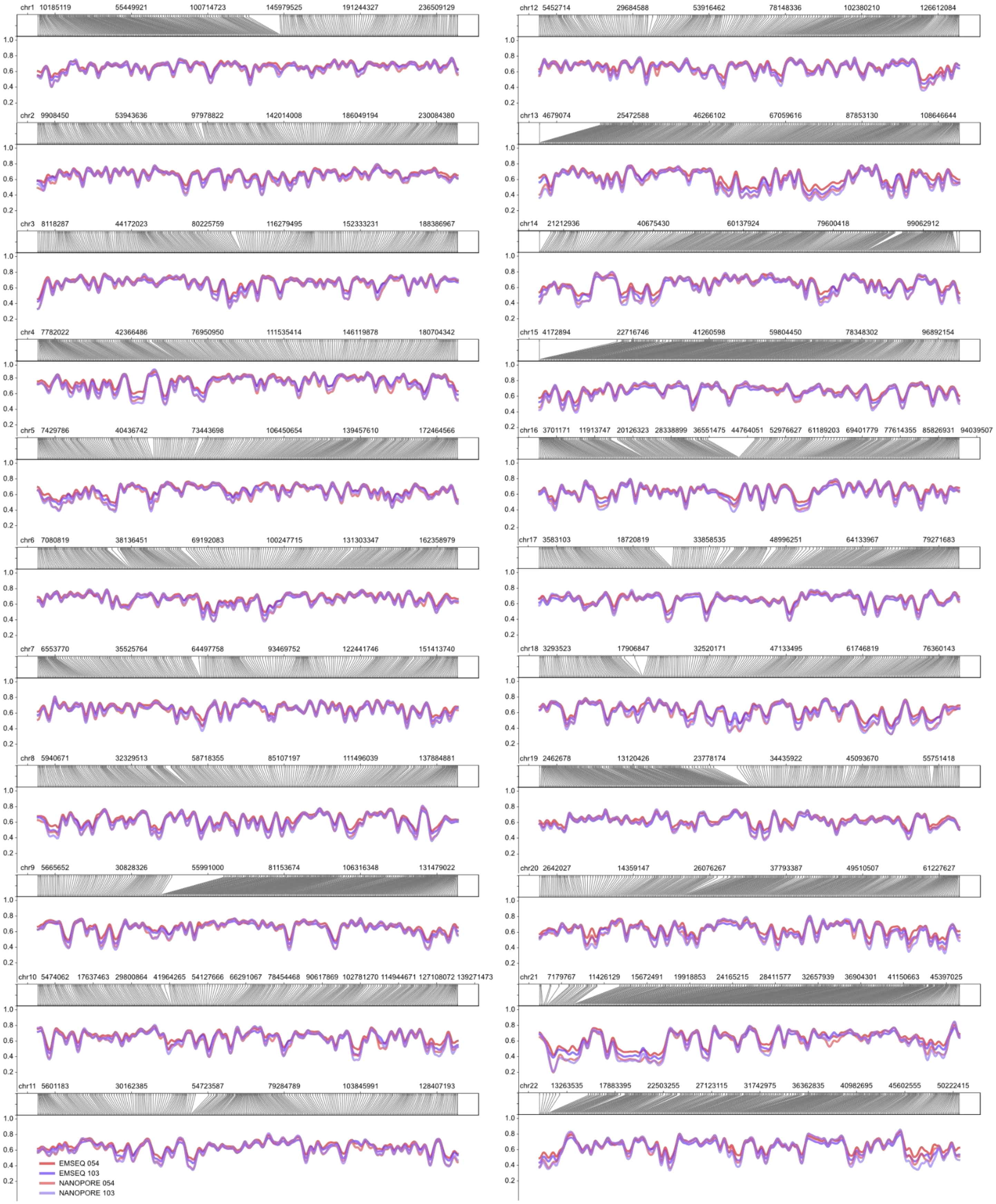

Supplemental Figure 2: Genome-wide placental genome methylation profiles compared between Nanopore and EM-seq output for two samples (054 and 103). Each panel shows genome coordinate space translated to CpG coordinate space, chromosomes are not scaled by length.

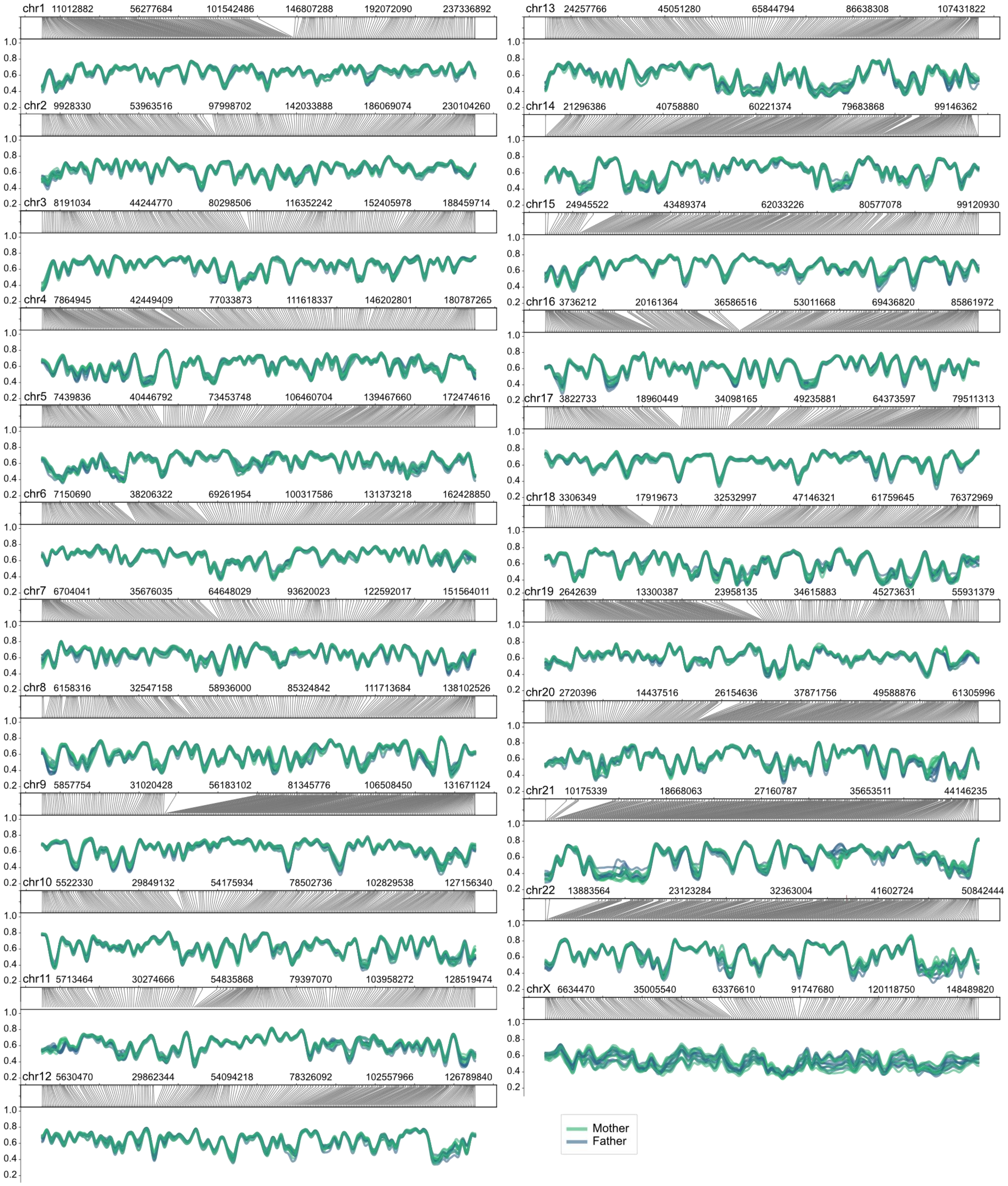

Supplemental Figure 3: Haplotype-resolved methylation by parent-of-origin. Maternal and paternal alleles are broadly similar with the exception of the X chromosome (see Supplemental Figure 3).

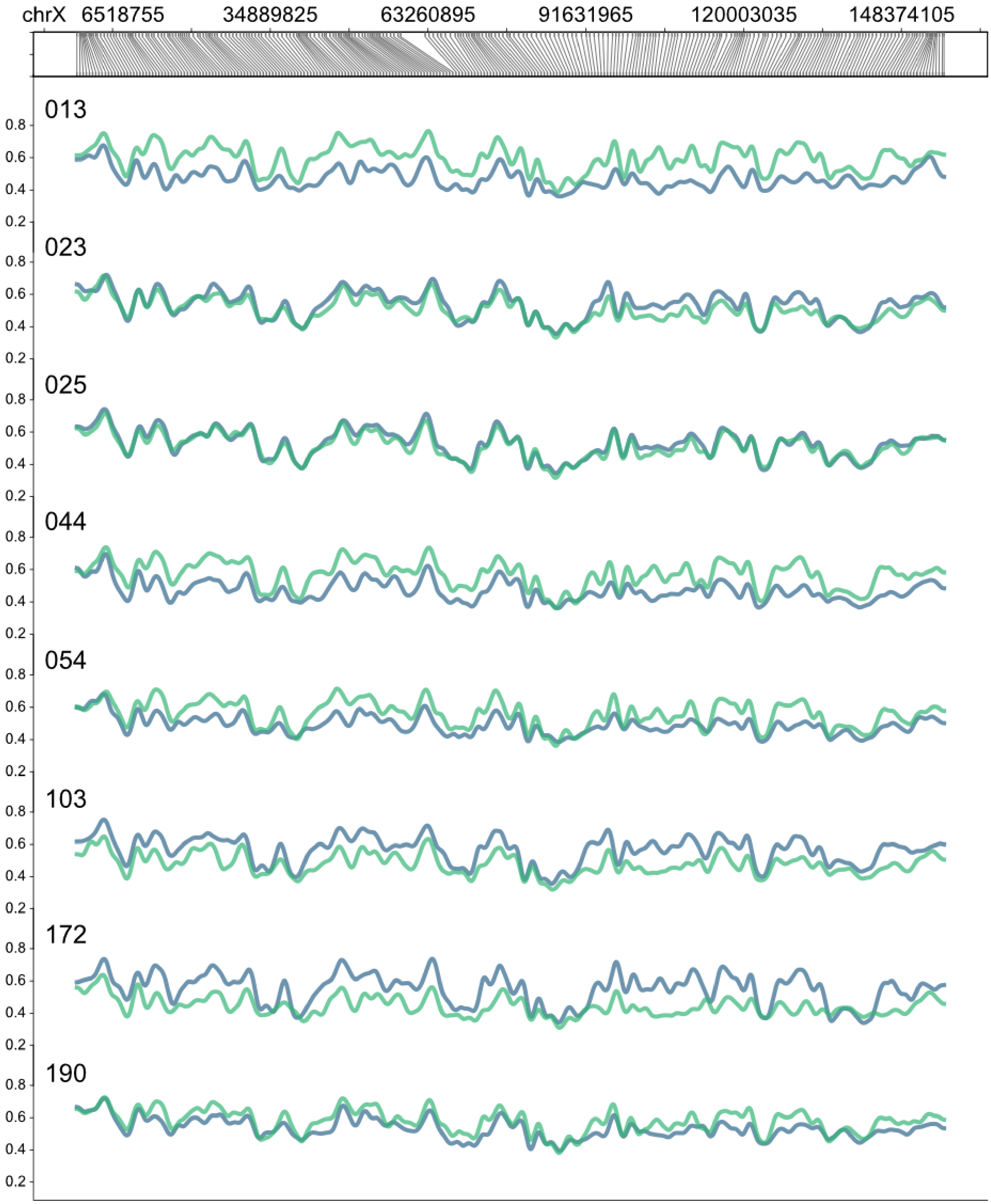

Supplemental Figure 4: Comparison of maternal (green) and paternal (blue) methylation profiles on chromosome X. In some samples the maternal chromosome is generally more methylated (013, 044, 045, 190), in some the paternal chromosome is more methylated (103, 172) and in others methylation levels are comparable (023, 025). As noted, this is likely the result of the clonal development of placental tissue.

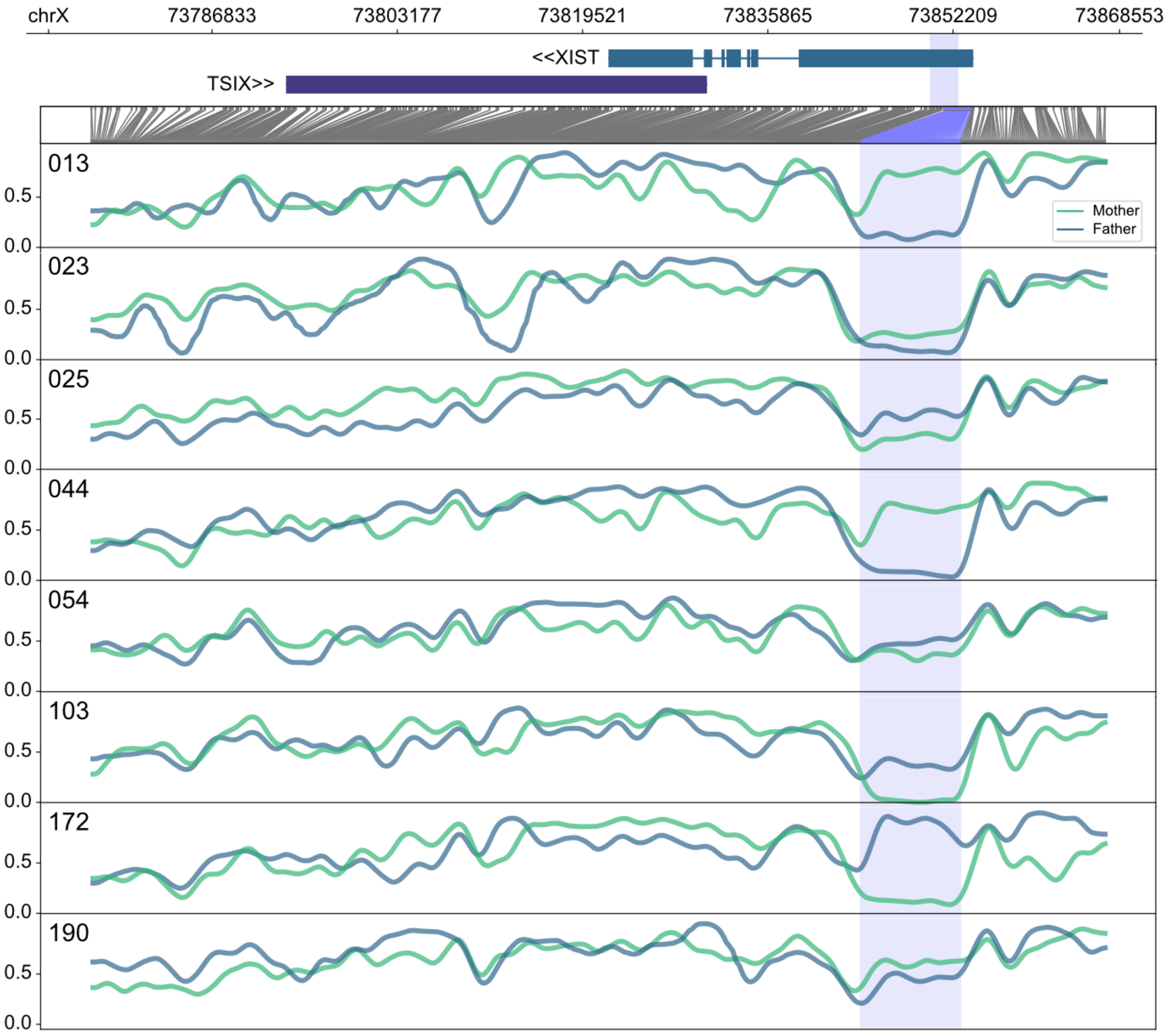

Supplemental Figure 5: Allele-specific CpG methylation of XIST Promoter region. Related to Supplemental Figure 3.

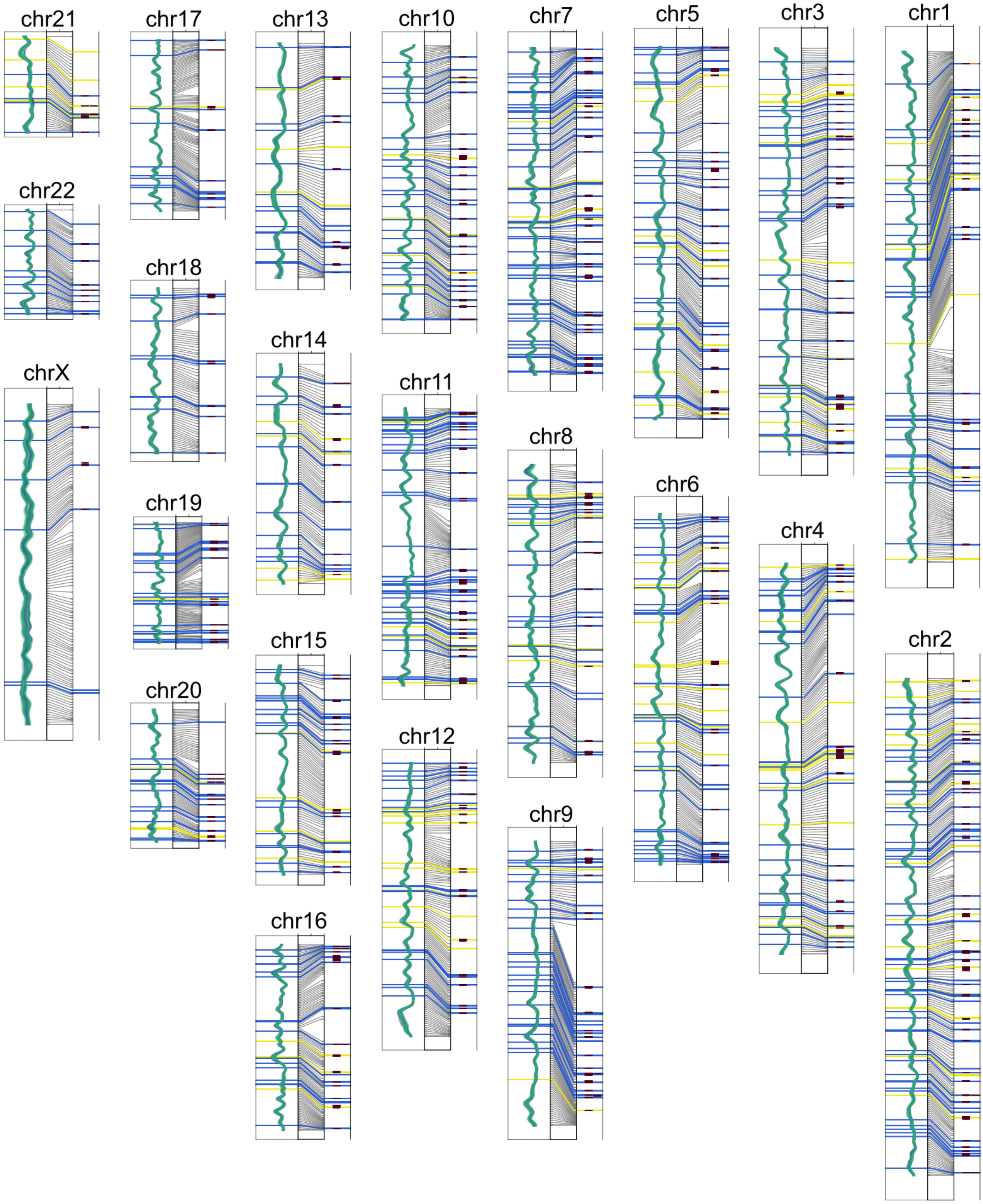

Supplemental Figure 6: Novel and previously described DMRs distributed across the genome. Novel DMRs are indicated as yellow highlights and those previously described are highlighted in blue. Associated protein-coding genes are included in the rightmost track and the parent-specific methylation profiles are included on the left track of each chromosome plot with the translation between genome and CpG coordinate space in between.

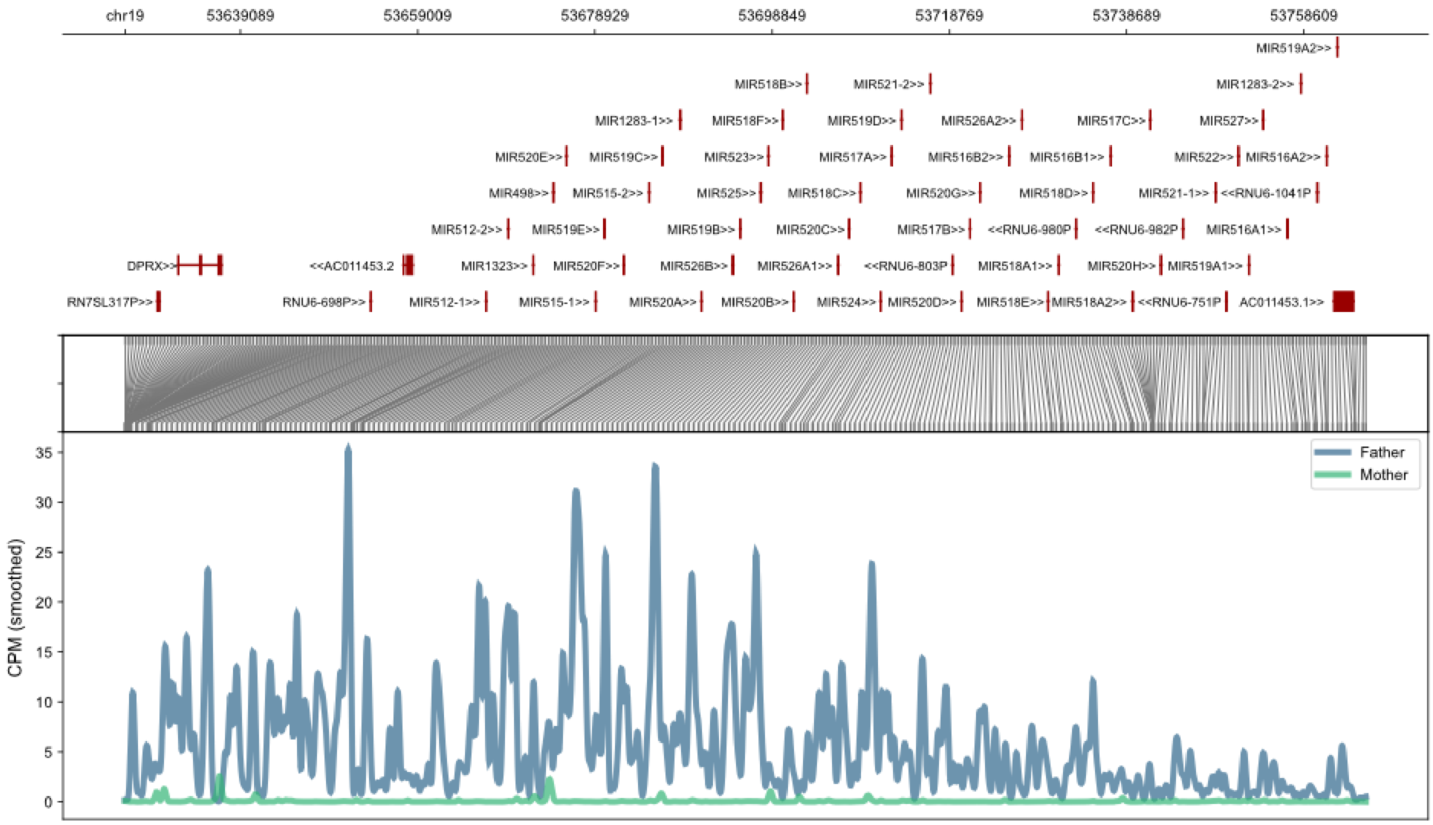

Supplemental Figure 7: Allele-specific expression profiles of the C19MC region containing many paternally-imprinted miRNAs. The top panel shows coordinates and gene locations, followed by a translation from genome coordinate space into expressed coordinate space.

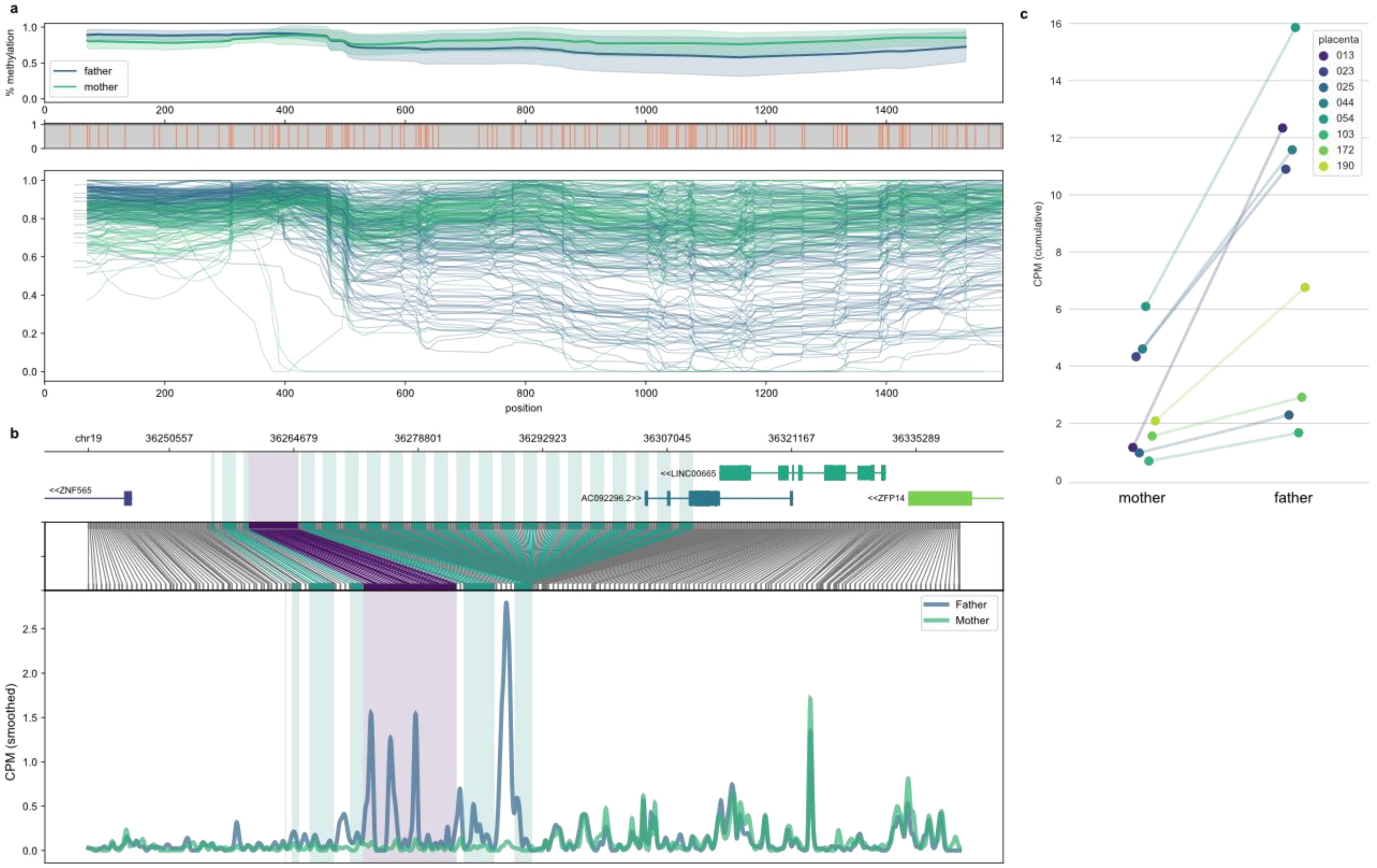

Supplemental Figure 8: Panel (a) shows the parent-specific methylation profile of each SST1 repeat in the left chr19 SST1 array, with the averaged methylation plot on top. The allele-specific expression detectable at the SST1 array is shown in panel (b), with the caveat that allele-specific expression is only apparent over informative variants which are few in number across this highly repetitive locus. The purple highlight indicates the position of the HERVH element that seems to be driving allele-specific expression across the locus.

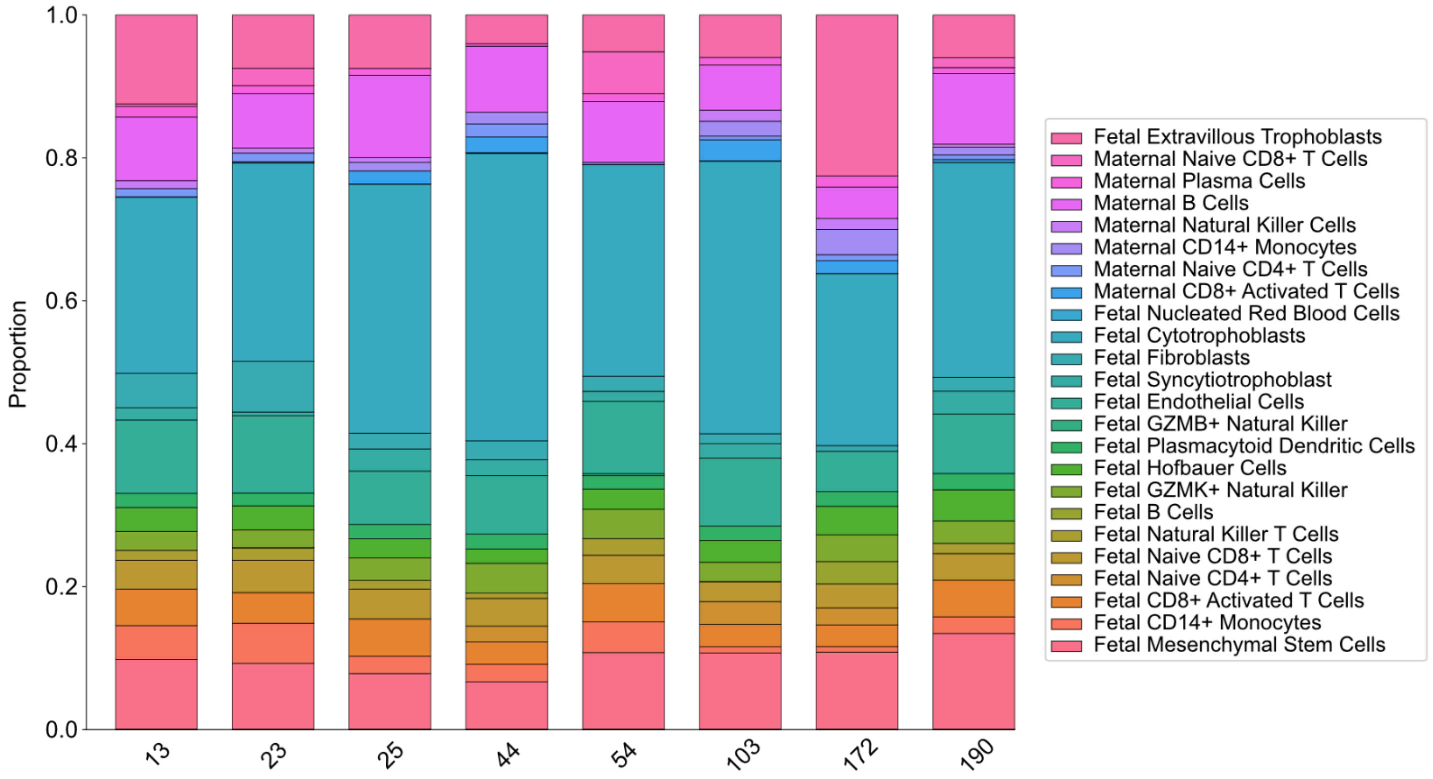

Supplemental Figure 9: Inference of placental cell type composition based on bulk RNA-seq via CIBERSORTx.

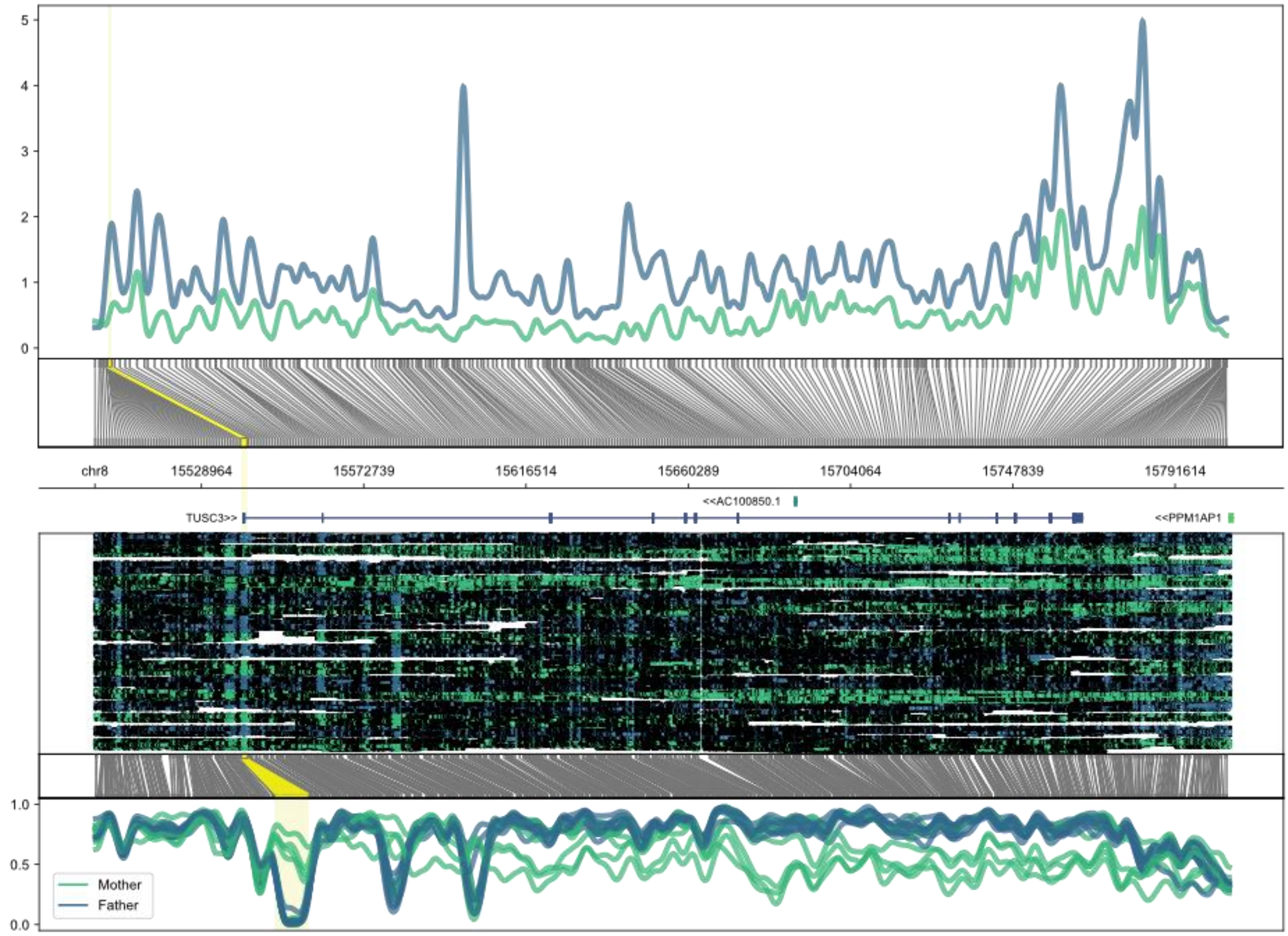

Supplemental Figure 10: Polymorphic allele-specific methylation (bottom panel) and paternal-biased expression (top panel) of TUSC3. The DMR location is highlighted (yellow) across the panels.

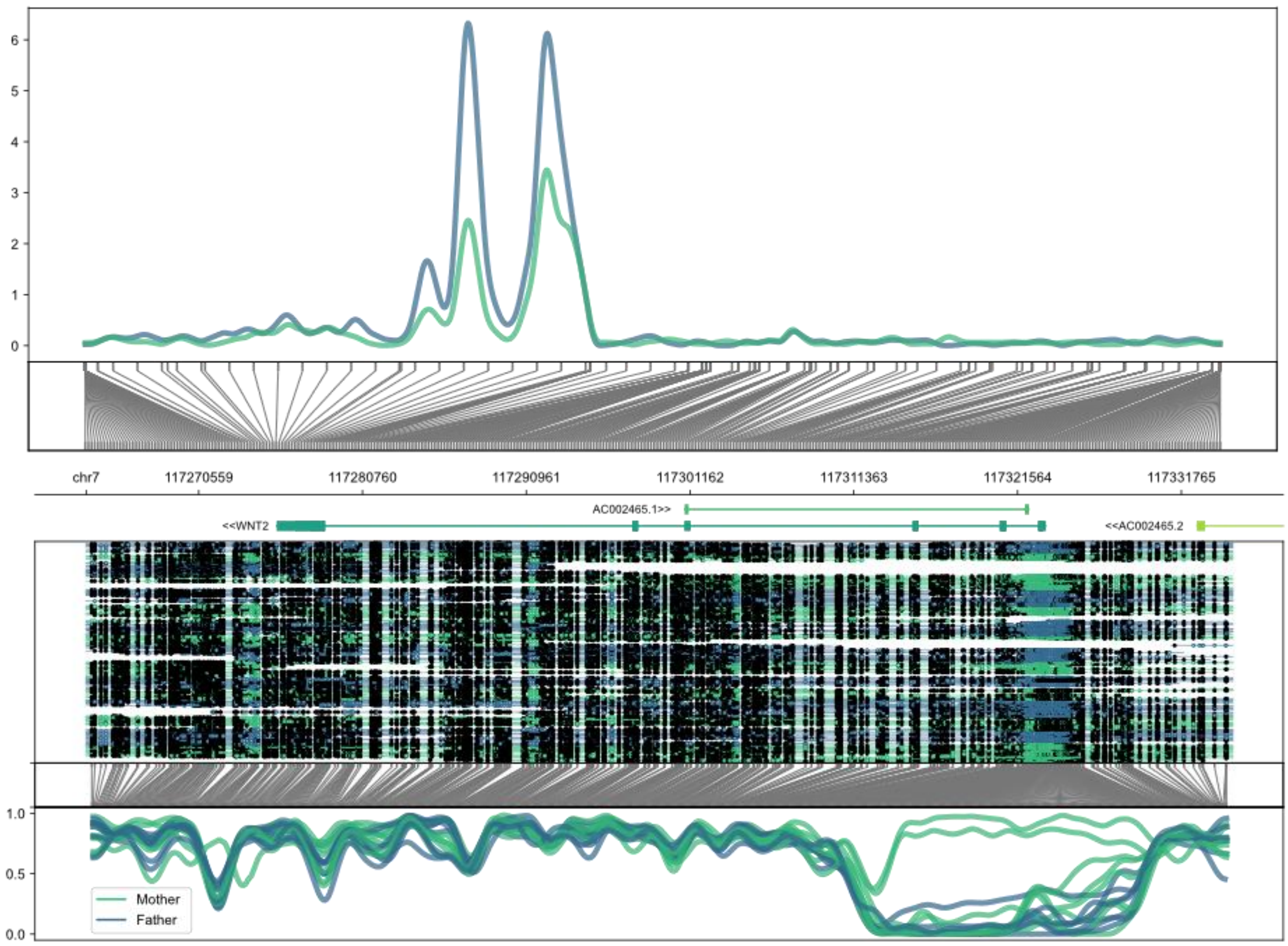

Supplemental Figure 11: Polymorphic allele-specific methylation (bottom panel) and paternal-biased expression (top panel) of WNT2.

Supplementary Data Legends

Supplementary Data 1: Statistics of related to sequence data generated for this study for **(a)** whole-genome sequencing data including phasing statistics where applicable, **(b)** RNA-sequencing including phasing statistics, and **(c)** Enzymatic methylation sequencing (EM-seq).

Supplementary Data 2: Estimation of maternal contamination in placental genomic DNA based on alleleic balance (Variant allele fraction, VAF) of heterozygous SNPs inherited maternally and paternally. Contamination would manifest as deviation from the expected heterozygous VAF = 0.5, which was not observed.

Supplementary Data 3: Summary information related to differentially methylated regions (DMRs) between maternal and paternal chromosomes. Included information (column descriptions) is detailed in the “Legend” tab.

Supplementary Data 4: Evaluation of DMRs detected from nanopore sequence data in EM-seq data for two placental samples (054 and 103). DMRs included in this table were covered by sufficient methylation calls (methylated + unmethylated CpG count > 20) in one or the other of the two EM-seq samples, DMRs present in Supplementary Data 3 but absent from this table were not detected above this threshold in either of the EM-seq samples. Fisher’s exact test (FET) was used to assess differential methylated vs unmethylated CpGs for each covered DMR. The FET odds ratio (F) and uncorrected P-value (P) is given for each sample where the DMR was covered.

Supplementary Data 5: Differential expression between maternal and paternal alleles. Columns are output from edgeR glmLRT function. logFC: log fold change, logCPM: log counts-per-million, LR: likelihood ratio, PValue: P-value from likelihood ratio test, FDR: corrected P-value.

Supplementary Data 6: Somatic mutations detected in placental samples. SNPs and INDELs **(a)** related to Figure 6. Overall count (Total, SNV, Indel) are represented by points in the FIgure 6 swarm plots, those with significantly low VAF (binomial test) are shown in the “lowVAF” columns and are represented as red points in Figure 6. Details of somatic structural variants are provided in **(b)** and further details concerning the putative somatic L1 insertion detected are shown in **(c)**.

Supplementary Data 7: Aggregated clinical variables pertaining to samples used in this study. Mean, median, minimum, and maximum values are given for each numeric variable and a summary of categorical variables is shown below.
