# Supplementary material for: An allele-resolved nanopore-guided tour of the human placental methylome
Source: Nat Commun. 2025 Nov 24;16:10358. doi: 10.1038/s41467-025-65337-3 (PMC12644668; doi:10.1038/s41467-025-65337-3)
Supplement: Supplementary file 9 — Reporting Summary [file 41467_2025_65337_MOESM9_ESM.pdf]

nature portfolio

Corresponding author(s): Ewing

Last updated by author(s): 30/09/2025

Reporting Summary

Nature Portfolio wishes to improve the reproducibility of the work that we publish. This form provides structure for consistency and transparency in reporting. For further information on Nature Portfolio policies, see our Editorial Policies and the Editorial Policy Checklist.

Statistics

For all statistical analyses, confirm that the following items are present in the figure legend, table legend, main text, or Methods section.

n/a	Confirmed	
☐	☒	The exact sample size (*n*) for each experimental group/condition, given as a discrete number and unit of measurement
☐	☒	A statement on whether measurements were taken from distinct samples or whether the same sample was measured repeatedly
☐	☒	The statistical test(s) used AND whether they are one- or two-sided<br>*Only common tests should be described solely by name; describe more complex techniques in the Methods section.*
☒	☐	A description of all covariates tested
☒	☐	A description of any assumptions or corrections, such as tests of normality and adjustment for multiple comparisons
☐	☒	A full description of the statistical parameters including central tendency (e.g. means) or other basic estimates (e.g. regression coefficient) AND variation (e.g. standard deviation) or associated estimates of uncertainty (e.g. confidence intervals)
☐	☒	For null hypothesis testing, the test statistic (e.g. *F*, *t*, *r*) with confidence intervals, effect sizes, degrees of freedom and *P* value noted<br>*Give P values as exact values whenever suitable.*
☒	☐	For Bayesian analysis, information on the choice of priors and Markov chain Monte Carlo settings
☒	☐	For hierarchical and complex designs, identification of the appropriate level for tests and full reporting of outcomes
☐	☒	Estimates of effect sizes (e.g. Cohen's *d*, Pearson's *r*), indicating how they were calculated

Our web collection on statistics for biologists contains articles on many of the points above.

Software and code

Policy information about availability of computer code

Data collection

For nanopore data, quantification of methylation and visualisations were created using methylartist 1.0.6 (Cheetham et al. 2022). EM-seq data was quality controlled and processed using BISCUIT 1.4.0 (Zhou et al. 2024). Differential methylation analysis was carried out using DSS 2.54.0.

Data analysis

Methylation:

For nanopore data, quantification of methylation and visualisations were created using methylartist 1.0.6 (Cheetham et al. 2022). EM-seq data was quality controlled and processed using BISCUIT 1.4.0 (Zhou et al. 2024). Differential methylation analysis was carried out using DSS 2.54.0. Quantification of methylation and visualisations were created using methylartist 1.0.6 (Cheetham et al. 2022).

RNA-seq:

Reads were aligned to the reference genome (hg38, GATK Resource Bundle) using STAR 2.7.10a (Dobin et al. 2012), duplicate reads were marked via samblaster 0.1.26 (Faust and Hall 2014). Aligned reads were assessed against quality control metrics using rnaseqc 2.4.2 (Graubert et al. 2021) and CollectRnaSeqMetrics from picard 2.27.5. Reads were phased using whatshap-phased variants as per long read DNA sequencing. Reads were counted against DMR locations and against Ensembl gene build 106 using featureCounts from Rsubread 4.1.2. Assessment of cell type composition was carried out via the CIBERSORTx docker container (Newman et al. 2019).

Mutation calling:

To detect germline variants used for phasing, variants for each trio were called jointly with GATK 4.1.9 HaplotypeCaller and quality scores

were recalibrated using GATK VQSR. Putative de novo and somatic variants were detected using GATK Mutect2 (Benjamin et al. 2019), with gnomAD 3.1.2 (Karczewski et al. 2020) as a germline resource.

Structural variants were detected from short-read data via delly v1.1.6 (Rausch et al. 2012) on a per-trio basis, filtered for somatic variants in the placenta sample, genotyped against the whole cohort and further filtered for somatic variants versus the whole-cohort genotyping results. SVs from long-read data were detected via sniffles2 version 2.0.4 (Sedlazeck et al. 2018) using the –non-germline and –phased options.

Variants called from Illumina along with pedigree information (trios) were phased using nanopore reads via WhatsHap v1.4 (Patterson et al. 2015).

Specific commands, scripts, and data for generating main and supplementary figures are available at https://github.com/adamewing/KindlovaByrne2025

For manuscripts utilizing custom algorithms or software that are central to the research but not yet described in published literature, software must be made available to editors and reviewers. We strongly encourage code deposition in a community repository (e.g. GitHub). See the Nature Portfolio guidelines for submitting code & software for further information.

Data

Policy information about availability of data

All manuscripts must include a data availability statement. This statement should provide the following information, where applicable:

- Accession codes, unique identifiers, or web links for publicly available datasets
- A description of any restrictions on data availability
- For clinical datasets or third party data, please ensure that the statement adheres to our policy

Data Availability
All sequence data generated in this study have been deposited at the EGA with accession EGAD50000001850 via controlled (mediated) access due to human research ethics requirements. We endeavor to respond to access requests within two weeks. to Processed data is available in the form of allele-specific methylation levels for all placental samples as indexed bedMethyl format at https://doi.org/10.6084/m9.figshare.28593515.v1. Additional supplemental data is available at https://doi.org/10.6084/m9.figshare.22589635.v1.
Nanopore sequence data from Heart, Liver, and Hippocampus tissues (Ewing et al. 2020) was obtained from SRA Bioproject PRJNA629858 [https://www.ncbi.nlm.nih.gov/bioproject/PRJNA629858]. Nanopore sequence data from NA19240 (De Coster et al. 2019) was obtained via ENA accession PRJEB26791 [https://www.ebi.ac.uk/ena/browser/view/PRJEB26791]. MCF-7 sequence data (Cheetham et al. 2022) was obtained from SRA Bioproject PRJNA748257. Placenta single-cell sequencing read counts (Campbell et al. 2023) was obtained from GEO accession GSE182381.
Code Availability
Primary analyses were conducted via Methylartist (Cheetham et al. 2022). Scripts for using methylartist output in DSS are included in the 'scripts' directory in the methylartist repository (https://github.com/adamewing/methylartist). Specific methylartist commands, additional scripts for generating figures, and supporting data specific to this study are available at https://github.com/adamewing/KindlovaByrne2025 (https://doi.org/10.5281/zenodo.15532400) and at https://doi.org/10.6084/m9.figshare.28636532.v1.

Research involving human participants, their data, or biological material

Policy information about studies with human participants or human data. See also policy information about sex, gender (identity/presentation), and sexual orientation and race, ethnicity and racism.

Reporting on sex and gender	All placental samples were female as assigned at birth.
Reporting on race, ethnicity, or other socially relevant groupings	Samples represent a cross-selection of the Queensland Family Cohort population and are not selected according to any particular ethnicity or other group.
Population characteristics	As above, samples are form the Queensland Family Cohort (QFC) and are representative of uncomplicated pregnancies presenting at Mater Mother's Hospital over the last 5 years in Brisbane, Queensland, Australia.
Recruitment	For the QFC protocol, including recruitment criteria, please see https://bmjopen.bmj.com/content/11/6/e044463
Ethics oversight	Human research ethics approval for this study was granted by the Mater Misericordiae Limited HREC (HREC/MML/73929)

Note that full information on the approval of the study protocol must also be provided in the manuscript.

Field-specific reporting

Please select the one below that is the best fit for your research. If you are not sure, read the appropriate sections before making your selection.

☒ Life sciences ☐ Behavioural & social sciences ☐ Ecological, evolutionary & environmental sciences

For a reference copy of the document with all sections, see nature.com/documents/nr-reporting-summary-flat.pdf

Life sciences study design

All studies must disclose on these points even when the disclosure is negative.

Sample size	Exploratory study, sample size was chosen to balance financial feasibility with replication
Data exclusions	not applicable
Replication	Findings replicated against known differentially methylated regions (Supplemental Table 3) and against orthogonal data type (Nanopore compared to EM-seq). Genomic variants are replicated across long-read and short-read methods.
Randomization	not applicable
Blinding	not applicable

Reporting for specific materials, systems and methods

We require information from authors about some types of materials, experimental systems and methods used in many studies. Here, indicate whether each material, system or method listed is relevant to your study. If you are not sure if a list item applies to your research, read the appropriate section before selecting a response.

Materials & experimental systems

n/a	Involved in the study
☒	☐ Antibodies
☒	☐ Eukaryotic cell lines
☒	☐ Palaeontology and archaeology
☒	☐ Animals and other organisms
☒	☐ Clinical data
☒	☐ Dual use research of concern
☒	☐ Plants

Methods

n/a	Involved in the study
☒	☐ ChIP-seq
☒	☐ Flow cytometry
☒	☐ MRI-based neuroimaging

Plants

Seed stocks	*Report on the source of all seed stocks or other plant material used. If applicable, state the seed stock centre and catalogue number. If plant specimens were collected from the field, describe the collection location, date and sampling procedures.*
Novel plant genotypes	*Describe the methods by which all novel plant genotypes were produced. This includes those generated by transgenic approaches, gene editing, chemical/radiation-based mutagenesis and hybridization. For transgenic lines, describe the transformation method, the number of independent lines analyzed and the generation upon which experiments were performed. For gene-edited lines, describe the editor used, the endogenous sequence targeted for editing, the targeting guide RNA sequence (if applicable) and how the editor was applied.*
Authentication	*Describe any authentication procedures for each seed stock used or novel genotype generated. Describe any experiments used to assess the effect of a mutation and, where applicable, how potential secondary effects (e.g. second site T-DNA insertions, mosiacism, off-target gene editing) were examined.*
